# A Mobile App for the Self-Management of Type 1 Diabetes Among Adolescents: A Randomized Controlled Trial

**DOI:** 10.2196/mhealth.7336

**Published:** 2017-06-19

**Authors:** Shivani Goyal, Caitlin A Nunn, Michael Rotondi, Amy B Couperthwaite, Sally Reiser, Angelo Simone, Debra K Katzman, Joseph A Cafazzo, Mark R Palmert

**Affiliations:** ^1^ Centre for Global eHealth Innovation Techna Institute University Health Network Toronto, ON Canada; ^2^ Institute of Biomaterials and Biomedical Engineering University of Toronto Toronto, ON Canada; ^3^ Division of Endocrinology The Hospital for Sick Children Toronto, ON Canada; ^4^ School of Kinesiology & Health Science York University Toronto, ON Canada; ^5^ Trillium Health Partners Toronto, ON Canada; ^6^ Research Institute The Hospital for Sick Children Toronto, ON Canada; ^7^ Division of Adolescent Medicine Department of Pediatrics The Hospital for Sick Children Toronto, ON Canada; ^8^ Institute of Health Policy, Management and Evaluation Dalla Lana School of Public Health University of Toronto Toronto, ON Canada; ^9^ Departments of Paediatrics and Physiology University of Toronto Toronto, ON Canada

**Keywords:** diabetes mellitus, mobile phone, cell phones, mobile applications, behavior change, blood glucose, self-management, self-care, adolescent, gamification

## Abstract

**Background:**

While optimal blood glucose control is known to reduce the long-term complications associated with type 1 diabetes mellitus, adolescents often struggle to achieve their blood glucose targets. However, their strong propensity toward technology presents a unique opportunity for the delivery of novel self-management interventions. To support type 1 diabetes self-management in this population, we developed the diabetes self-management app *bant*, which included wireless blood glucose reading transfer, out-of-range blood glucose trend alerts, coaching around out-of-range trend causes and fixes, and a point-based incentive system.

**Objective:**

The primary objective was to evaluate *bant* ’s effect on hemoglobin A_1c_ (HbA_1c_) through a randomized controlled trial (RCT). Secondary measures (eg, self-monitoring of blood glucose [SMBG]) were also collected to assess *bant* ’s impact on the self-management behaviors of adolescents with type 1 diabetes.

**Methods:**

We enrolled 92 adolescents into a 12-month RCT, with 46 receiving usual care and 46 receiving usual care plus *bant*. Clinical outcome data were collected at quarterly research visits via validated tools, electronic chart review, glucometer downloads, and semistructured interviews. App satisfaction was assessed at 6 and 12 months, and at trial end, users ranked *bant* components based on perceived usefulness. Mobile analytics captured frequency of blood glucose uploads, which were used to categorize participants into high, moderate, low, or very low engagement levels.

**Results:**

Linear mixed models showed no changes in primary and secondary clinical outcomes. However, exploratory regression analysis demonstrated a statistically significant association between increased SMBG and improved HbA_1c_ in the intervention group. For a subgroup of *bant* users taking SMBG ≥5 daily, there was a significant improvement in HbA_1c_ of 0.58% (*P*=.02), while the parallel subgroup in the control arm experienced no significant change in HbA_1c_ (decrease of 0.06%, *P=*.84). Although app usage did diminish over the trial, on average, 35% (16/46 participants) were classified as moderately or highly engaged (uploaded SMBG ≥3 days a week) over the 12 months.

**Conclusion:**

Although primary analysis of clinical outcomes did not demonstrate differences between the *bant* and control groups, exploratory analysis suggested that *bant* may positively impact the use of SMBG data and glycemic control among youth. The next generation of *bant* will aim to remove barriers to use, such as deploying the app directly to personal devices instead of secondary research phones, and to explore the utility of integrating *bant* into routine clinical care to facilitate more frequent feedback. Future evaluations of mHealth apps should consider more robust research tools (eg, ResearchKit) and alternative RCT study designs to enable more rapid and iterative evaluations, better suited to the nature of rapidly evolving consumer technology.

**Trial Registration:**

ClinicalTrials.gov NCT01899274; https://clinicaltrials.gov/ct2/show/NCT01899274 (Archived by WebCite at http://www.webcitation.org/6qWrqF1yw)

## Introduction

Type 1 diabetes mellitus is among the most common chronic diseases affecting children, adolescents, and adults, with an increasing worldwide incidence of approximately 3% to 4% a year [1,2]. Optimizing blood glucose control is important for patients with type 1 diabetes, as improved control has been shown to reduce the incidence and severity of type 1 diabetes complications and diabetes-related mortality [3-6]. However, achieving optimal control requires intensive self-management, which can be challenging for patients to achieve. Adolescents, in particular, struggle with optimizing blood glucose control, with worldwide data indicating they consistently fail to meet their prescribed therapeutic targets [7,8].

Overall, advancements in the mechanism of insulin delivery (ie, insulin pump or multiple daily injections) has had a limited impact on glycemic control among youth [9,10]. Instead, research has suggested that self-care factors, such as targeted goal setting and improved self-monitoring of blood glucose (SMBG), along with educational models, may have a greater impact on health outcomes [11-13]. Given adolescents’ propensity for new technology, eHealth interventions may provide a unique opportunity to communicate with and motivate youth and thereby improve their diabetes management [14,15]. Teenagers are adopting new forms of technology quicker and in a more immersive way than any other age group, with the mobile phone becoming a primary communication tool for this demographic [16,17]. In 2015, it was reported that 88% of American teens either owned or had access to a mobile phone, up from 45% in 2004 [16,17].

Recently, the use of mHealth apps as a tool for improved diabetes self-management has proliferated, as illustrated by the number of diabetes apps available for download on the iOS App Store and Google Play [18-23]. While interest in this technology continues to rise, the clinical utility of these apps remains unclear [24]. Only a limited number of diabetes apps have completed rigorous evaluation and, to date, most studies have been conducted for the adult [25] and/or type 2 diabetes mellitus population [26,27]. How effective these apps are among adolescents with type 1 diabetes remains unknown.

Furthermore, many of the existing apps require manual entry of blood glucose values and focus primarily on the display of diabetes-related data, such as blood glucose readings, carbohydrate intake, and insulin doses [24,28]. However, recent reviews have demonstrated that very few of these apps use this information to provide users with personalized feedback, education, or motivation [28-30]. With clinical guidelines emphasizing the importance of individualized feedback and targeted education, failing to provide users with these features puts current apps at risk of simply mirroring paper-based tools, instead of being a means for behavior change and comprehensive self-management [31].

Therefore, the objective of this research was to design, develop, and evaluate *bant*, an app aimed to assist adolescents with the self-management of type 1 diabetes. In 2010-2011, a pilot version of *bant* was developed and evaluated through a 12-week pilot study (n=20) among adolescents with type 1 diabetes, aged 12-16 years, with hemoglobin A_1c_ (HbA_1c_) between 8% and 10%. Results showed an increase in daily SMBG by 50% (*P*=.006) and a high reported level of satisfaction, with 88% of respondents stating they would continue to use the system [32]. While use of *bant* led to improved self-management behaviors, the trial was not designed to assess effect on HbA_1c_. This paper reports the results of a 12-month randomized controlled trial (RCT) conducted to assess the effectiveness of an updated version of *bant* as a self-management tool for adolescents with type 1 diabetes (ClinicalTrials.gov NCT01899274; Multimedia Appendix 1 [33]).

## Methods

Adolescents with a diagnosis of type 1 diabetes, between the ages of 11 and 16 years, were randomly assigned to 1 of 2 groups: (1) the *bant* (intervention) group, or (2) the treatment as usual (control) group. Both groups were followed for a duration of 12 months.

### Ethical Approval

Before initiating the study, protocol approval was obtained from all site-specific ethical review boards and/or committees (The Hospital for Sick Children: #1000036524; University Health Network: #13-6237-BE; Trillium Health Partners: #619).

### Enrollment

We recruited participants from August 2013 to December 2014 from 2 pediatric endocrinology centers in Toronto, Ontario, Canada. The final study visit was completed in January 2016. Patients were eligible to participate if they (1) had a diagnosis of type 1 diabetes mellitus (as defined by Canadian Diabetes Association guidelines [31]) for 1 year or more, (2) were between the ages of 11 and 16 years, inclusive, at the time of enrollment, (3) had been followed at the current clinic for at least 6 months, and (4) had 2 of their 3 most recent HbA_1c_ readings (including the day of enrollment) between 8.0% and 10.5%. We selected this HbA_1c_ range in an attempt to identify patients who were struggling with their glycemic control, and for whom the use of a smartphone app alone might be an appropriate intervention. Given that *bant* was only offered in English at the time of recruitment, participants were excluded if they did not fluently speak and understand English. All participants and parents provided written, informed consent prior to participation.

### Sample Size

Sample size was determined based on a nominal 2-sided type I error rate of 5% and 80% power. Estimates of standard deviation in HbA_1c_ ranging from 0.50% to 0.75% were used to determine the minimum number of participants required to detect a clinically relevant (≥0.5%) change in HbA_1c_ levels [34-36]. Standard deviation estimations were consistent with the *bant* pilot study, which reported a baseline standard deviation of 0.55% in HbA_1c_ levels, and were also informed by longitudinal HbA_1c_ variation over 9 months in an independent sample of 13 patients. A final sample size of 92 participants (46 per intervention arm) allowed for a potential 25% dropout rate.

### Random Allocation

At enrollment, participants were assigned equally to an intervention or control arm using randomly allocated block sequences of 4 to 6 participants. To ensure equal distribution between arms, we stratified random allocation for treatment modality (insulin pump vs insulin injection), as well as study center (The Hospital for Sick Children vs Trillium Health Partners). The RCT was an unblinded, open-label study, as both the participants and those delivering the intervention were aware of allocation based on whether or not the *bant* system was received. In addition, clinical outcomes were not blinded, as they are part of a participant’s ongoing clinical care and diabetes monitoring regimen.

### Intervention

**Figure 1:**
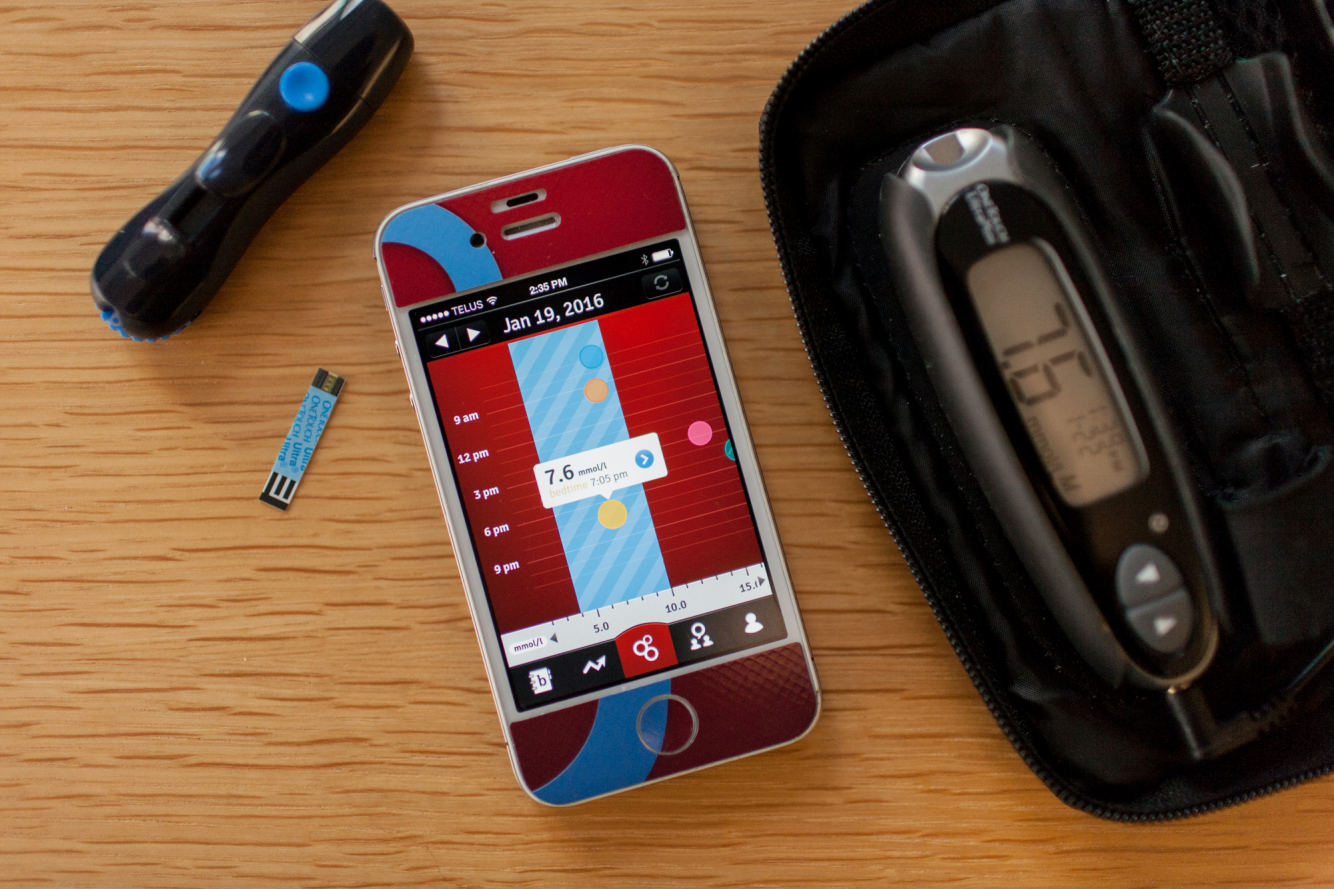
The intervention includes an iPhone 4S loaded with *bant*, as well as a Bluetooth adapter attached to the OneTouch UltraMini blood glucose meter. Circles represent individual readings at different times of the day, with the bedtime reading having been selected to display further information; the blue region represents a particular participant’s target blood glucose range. The different colors of the circles represent the different reading contexts (eg, breakfast readings are blue).

The initial design of *bant* was informed by insights gathered through qualitative ethnographic interviews conducted with adolescents living with type 1 diabetes and their families. In addition to patient input, we held focus group sessions with clinical staff who had experience managing type 1 diabetes and chronic disease among adolescents. Feedback from these sessions, as well as input from human factors specialists, informed the development of the pilot version of *bant*, which was then evaluated among 20 adolescents for 3 months. The initial focus group sessions, user-centered design, and evaluation of *bant* have been previously reported by Cafazzo et al [32]. Upon the completion of the pilot trial, we obtained feedback from participants, leading to further refinement of *bant* (Multimedia Appendix 2). It is important to note that, while the pilot version of *bant* was designed to incentivize more frequent SMBG, the updated version of *bant* included additional tactics that could potentially further facilitate improved HbA_1c_. Therefore, users were rewarded for taking SMBG but also for maintaining their blood glucose within their target range. Table 1 describes the key features of *bant* (for related screenshots, see Multimedia Appendix 2), and Figure 1 illustrates the system that the intervention group received.

**Table 1:** Key features of *bant*.

Feature	Description
Automatic Data Transfer	Blood glucose readings are wirelessly transferred from a Bluetooth-enabled blood glucose meter, using an adaptor (BluGlu), to *bant*.
Electronic Logbook	Current and past blood glucose readings categorized by context (eg, lunch) are displayed over multiple time frames (eg, 1 week, 1 month).
Trends	Percentages of readings in or out of target, per context, are displayed over various time frames (eg, over 30 days, 10% of breakfast readings were high).
Trend Wizard	Algorithm that detects and informs the user of consecutive out-of-range readings for the same context (eg, 3 consecutive high dinner readings) and prompts the user to identify the likely cause of the trend and potential fixes.
Reward System	Reward mechanism that awards points to encourage the following behaviors: (1) taking up to 5 readings per day, (2) getting readings in target range, (3) avoiding out-of-range trends, and (4) resolving any identified 3-day trends. Users can redeem their points for iTunes gift cards. *bant* also includes a leaderboard for users to see where they rank compared with their peers.
*banter*	A private social media community that allowed trial participants to communicate with each other.
Personal Health Record	Integration with TELUS health space, a secure personal health record that stored blood glucose data and enabled sharing with members of the care team.

### Study Protocol

Adolescents who met the inclusion criteria and provided informed consent were randomly allocated to receive either usual clinical care (control group) or usual clinical care plus *bant* (intervention group). For reference, usual clinic care was defined as the standard care all youth and adolescents with type 1 diabetes receive at their quarterly clinic visits, as dictated by Canadian Diabetes Association guidelines [31]. At baseline, those allocated to the intervention group received an iPhone 4S (Apple Inc, Cupertino, CA, USA) loaded with *bant*, a OneTouch UltraMini (Lifescan, Inc, Milpitas, CA, USA) blood glucose meter, and a Bluetooth adapter (BluGlu, a device developed by University Health Network for investigational purposes only) that allowed for wireless transmission of data from the blood glucose meter to *bant*. To facilitate independent use, all *bant* users received a standardized 1-hour tutorial at study enrollment, which included hardware setup, introduction to app features, username creation, and troubleshooting steps for potential issues. During this time, *bant* users also created a TELUS health space account (TELUS Health Solutions, Cambridge, ON, Canada), which allowed for remote and secure storage and backup of their blood glucose data. Control group participants also completed a baseline visit. However, they did not receive any study-related hardware from the research team. Both control and intervention participants received 2 movie theater passes in exchange for their effort and time during the baseline and all subsequent visits.

Baseline visits were followed by 3-, 6-, 9-, and 12-month research visits for all participants. All research visits coincided with the participant’s standard quarterly clinic visit; however, these visits were conducted separate from the clinic visit by trained research staff. Qualitative and quantitative data were collected at all follow-up visits via semistructured interviews, validated instruments, downloads of blood glucose meters, and electronic chart review. Halfway between each follow-up visit, we contacted participants in the *bant* group to ensure they were not experiencing any technical issues. No advice or communication around clinical care or their diabetes regimen was discussed with participants during these calls. At study end, the *bant* system was returned to research personnel.

### Primary Outcome Measures

The primary outcome of the study was change in HbA_1c_ (measured in percentage) from baseline to 12 months, between the intervention and control group. HbA_1c_ was measured during routine clinical blood work and accessed by research staff through electronic chart review. The primary research site (The Hospital for Sick Children) used a high-performance liquid chromatography assay (Bio-Rad Laboratories, Inc, Waterloo, ON, Canada) or an enzymatic assay (Abbott Laboratories, Ltd, North York, ON, Canada) to measure HbA_1c_, with internal quality control demonstrating excellent agreement among samples assayed by both methods (*r*>.99). The secondary site (Trillium Health Partners) measured HbA_1c_ using a point-of-care immunoassay (DCA 2000+, Siemens Healthcare Ltd, Oakville, ON, Canada) for all measurements.

### Secondary Outcome Measures

#### Hypoglycemic Events

The frequency of mild and severe hypoglycemic events was assessed as secondary measures of glycemic control. A severe hypoglycemic event was defined as any episode that required the assistance of another individual and a blood glucose reading below 2.8 mmol/L and/or a subsequent reversal of clinical symptoms with intake of oral carbohydrate, glucagon injection, or intravenous glucose [37]. A mild hypoglycemia event was defined as a blood glucose reading below 3.4 mmol/L.

The frequency of severe hypoglycemic events was self-reported by participants and/or their guardians during semistructured interviews conducted at baseline and all follow-up research visits. To capture the frequency of mild hypoglycemic events, the previous 50 days of blood glucose readings were downloaded from all available (study and/or personal) blood glucose meters and/or insulin pumps during the participant’s clinic appointment. All downloads were completed by trained staff using the manufacturer-provided electronic downloading programs, specific to each blood glucose meter or pump brand. In cases where not all hardware was available, participants estimated what percentage of their total blood glucose readings were on the devices they brought to clinic that day.

All individual readings below 3.4 mmol/L were recorded as an individual mild hypoglycemic event, except for low blood glucose readings taken within the same or consecutive-hour timeslots. Grouping contemporaneous readings together and counting them as a single episode ensured that a singular hypoglycemic event was not recorded multiple times.

#### Self-Monitoring of Blood Glucose

We measured the average number of daily SMBG using all data collected from the 50-day blood glucose meter and/or insulin pump printout(s). Each blood glucose reading was counted individually, except when taken within the same hour, in which case readings were grouped. Readings taken over a 2-hour period in apparent response to an initial low (<4.1 mmol/L) or high (>17.9 mmol/L) were also grouped together. Using the total counted readings and number of days collected, we calculated the average number of daily SMBG at baseline as well as each follow-up visit, and when warranted, corrected for the percentage of readings available as estimated by participants.

#### Self-Initiated Adjustments

We assessed the number of self-initiated adjustments made to a participant’s type 1 diabetes insulin regimen during qualitative interviews conducted at baseline and all follow-up visits to determine whether use of *bant* led participants to attempt to adjust their insulin regimens more frequently. A self-initiated adjustment was defined as a change made to the prescribed treatment regimen that was initiated by the participant and/or their guardian(s) and implemented between clinic appointments. Changes made to the regimen by the diabetes care team during a routine clinic visit were not included. Participants self-reported who (the participant and/or their parent[s]/guardian[s]) was responsible for initiating the adjustment(s), as well as whether the diabetes team had been contacted for input on the regimen change.

#### Validated Questionnaires

Validated instruments were used to capture quality of life, self-care, and management data. The Diabetes Quality of Life for Youth (DQOLY) questionnaire [38,39] and the Diabetes Family Responsibility Questionnaire (DFRQ) [40] were administered at 6- and 12-month visits; the Self Care Inventory [41-43] was administered at all time points. The Readiness to Change Survey (Multimedia Appendix 3, Participant Management Questionnaire) was captured at baseline to help characterize the study population [44,45]. All surveys were given to participants to complete independently during their research visit.

#### Satisfaction With bant

We assessed overall satisfaction with *bant* via qualitative interviews conducted at 6- and 12-month visits. On a 7-point Likert scale, ranging from 1 (very dissatisfied) to 7 (very satisfied), users were asked to rate overall satisfaction as well as satisfaction for 5 individual *bant* components: (1) trend wizard, (2) the leaderboard, (3) automatic blood glucose transfer, (4) *banter*, and (5) iTunes rewards. In addition to collecting satisfaction scores, we conducted semistructured interviews to gather qualitative feedback from *bant* users during their 6- and 12-month research visits. Users were asked to provide feedback on app features, content, and how *bant* influenced their overall type 1 diabetes management. They were also asked to list, in a free-form text field, the 3 most and least helpful features of *bant*.

#### Usage Data

We collected mobile usage data through a third-party service, Flurry (Yahoo, Sunnyvale, CA, USA), which tracked (1) the number of times users accessed *bant*, (2) how often they used certain features, and (3) the number of times users wirelessly uploaded data from their blood glucose meter.

### Statistical Analysis

Preliminary *t* tests and chi-square tests were used to determine if there were any statistically significant differences between the intervention and control groups for the primary and secondary outcomes and demographic characteristics at baseline. This step allowed us to ensure the comparability of both the intervention and control groups at baseline and to ensure that we did not have any chance imbalances that might have required further adjustment.

Subsequently, we used linear mixed models to determine whether there were any statistically significant differences between the treatment and control groups for the above-mentioned outcomes. As all outcomes of interest were continuous, a linear mixed-model approach provided a simple method to assess treatment efficacy while adjusting for the correlation of each participant over time (using a random effect). Moreover, this approach is more powerful than a repeated-measures analysis of variance (ANOVA), as it allows participants with missing values at 1 or more time points to contribute some information to the analysis, while a repeated-measures ANOVA requires the availability of data at all time points for each participant [46]. We examined each outcome graphically to determine whether the data were normally distributed. All outcomes were approximately normally distributed, with the exception of the number of mild hypoglycemic events, which appeared to be somewhat skewed. However, linear mixed models have the ability to assess data that are not normally distributed and remain robust, as long as the sample size is large [47]. As a result of the large sample size and graphical appearances of normality, this assumption appeared reasonable.

Secondary analyses relied on comparison between groups at the primary end point of 12 months using 2-sample *t* tests or chi-square tests. Moreover, additional exploratory univariate regression analyses examined the impact of SMBG on clinical outcomes for those who were taking SMBG 5 or more times per day at 12 months within both the intervention (n=8) and control (n=5) groups. Although this is a very small subgroup, it provides some insight into the potential role of *bant* in controlling diabetes for those participants who are engaged and actively monitoring their blood glucose levels. Due to small sample sizes, adjusting for other confounding variables was not possible. Additionally, we used exploratory analyses, including chi-square tests, 2-sample *t* tests, and regression analyses, to evaluate the effectiveness of *bant* in subgroups based on insulin regimen (insulin pump vs insulin injections) and baseline HbA_1c_ levels (≥9.0% vs <9.0%). Finally, usage and satisfaction data were also summarized for exploratory purposes. All statistical analyses were performed using SAS software version 9.4 for Windows (SAS Institute) Results were considered statistically significant at the *P* ≤.05 level, and all reported results are 2-tailed.

## Results

### Study Population

**Figure 2:**
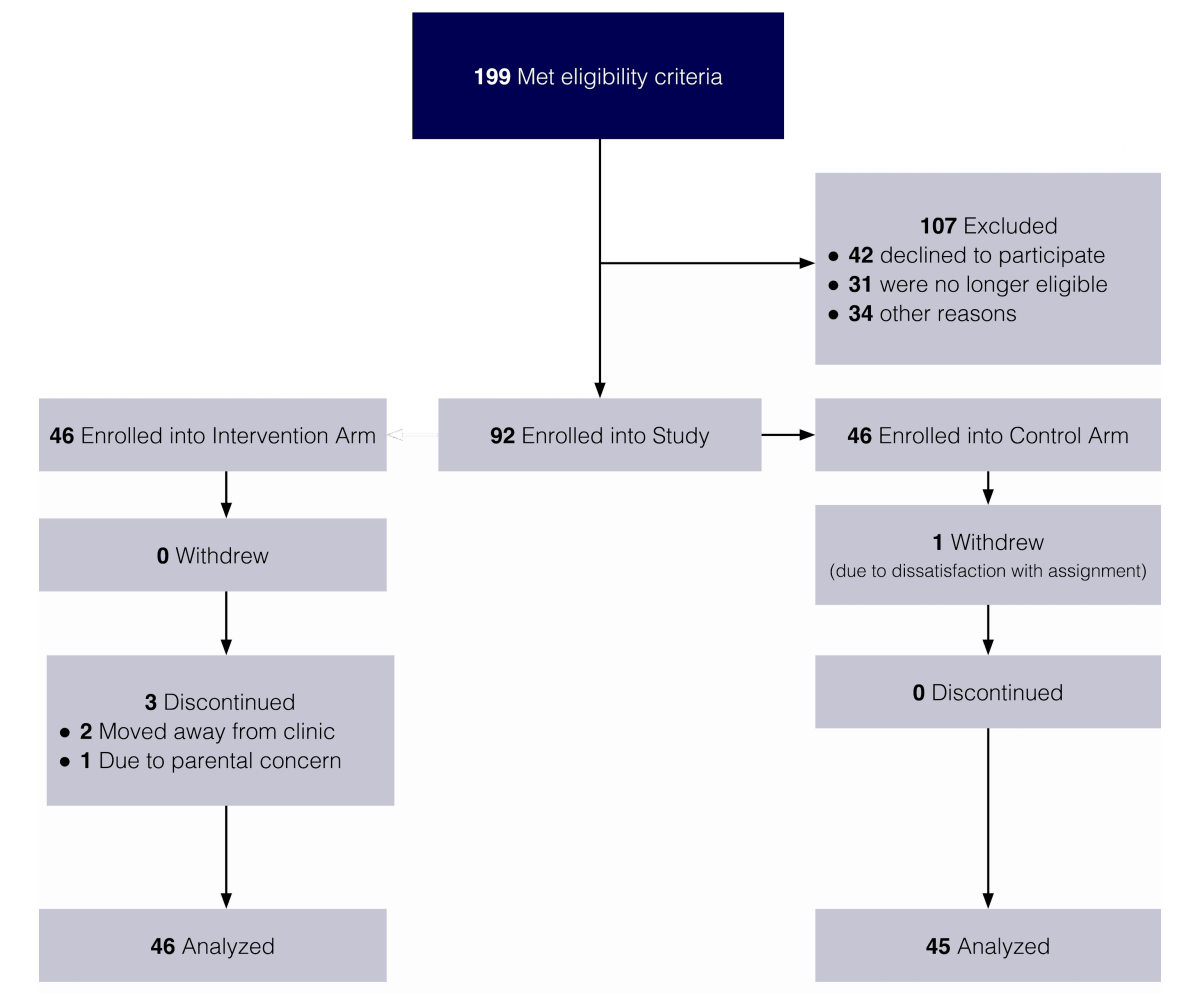
Participant enrollment.

Using the study inclusion criteria, we identified eligible participants from clinical databases and enrolled them sequentially until recruitment targets were met. Through this process, 199 eligible patients were identified; 42 patients declined to participate, 31 patients no longer met eligibility criteria, and 34 patients were excluded for other reasons, including planning to change clinics within the study time frame, having recently switched insulin regimens, and participating in another study with similar outcome measures. As Figure 2 shows, a total of 92 participants were enrolled and randomly allocated into the study.

Table 2 summarizes the demographic characteristics of the participants at baseline. There were no significant differences between the 2 groups in any of the measured characteristics, nor were there significant differences between the groups with respect to the readiness to change domains.

**Table 2:** Baseline characteristics of intervention and control groups.

Characteristics	Treatment group (n=46)	Control group (n=46)	*P* value
Sex (male/female), n	21/25	20/26	>.99
Age at baseline in years, mean (SD)	14.1 (1.7)	13.9 (1.5)	.54
Age at diagnosis in years, mean (SD)	7.1 (3.6)	7.4 (3.3)	.71
Duration of type 1 diabetes mellitus in years, mean (SD)	7.1 (3.2)	6.6 (3.2)	.48
Insulin regimen (pump/injection), n	23/23	22/24	.84
Hemoglobin A_1c_ in %, mean (SD)	8.96 (0.7)	8.92 (0.6)	.77

### Clinical Outcomes

**Figure 3:**
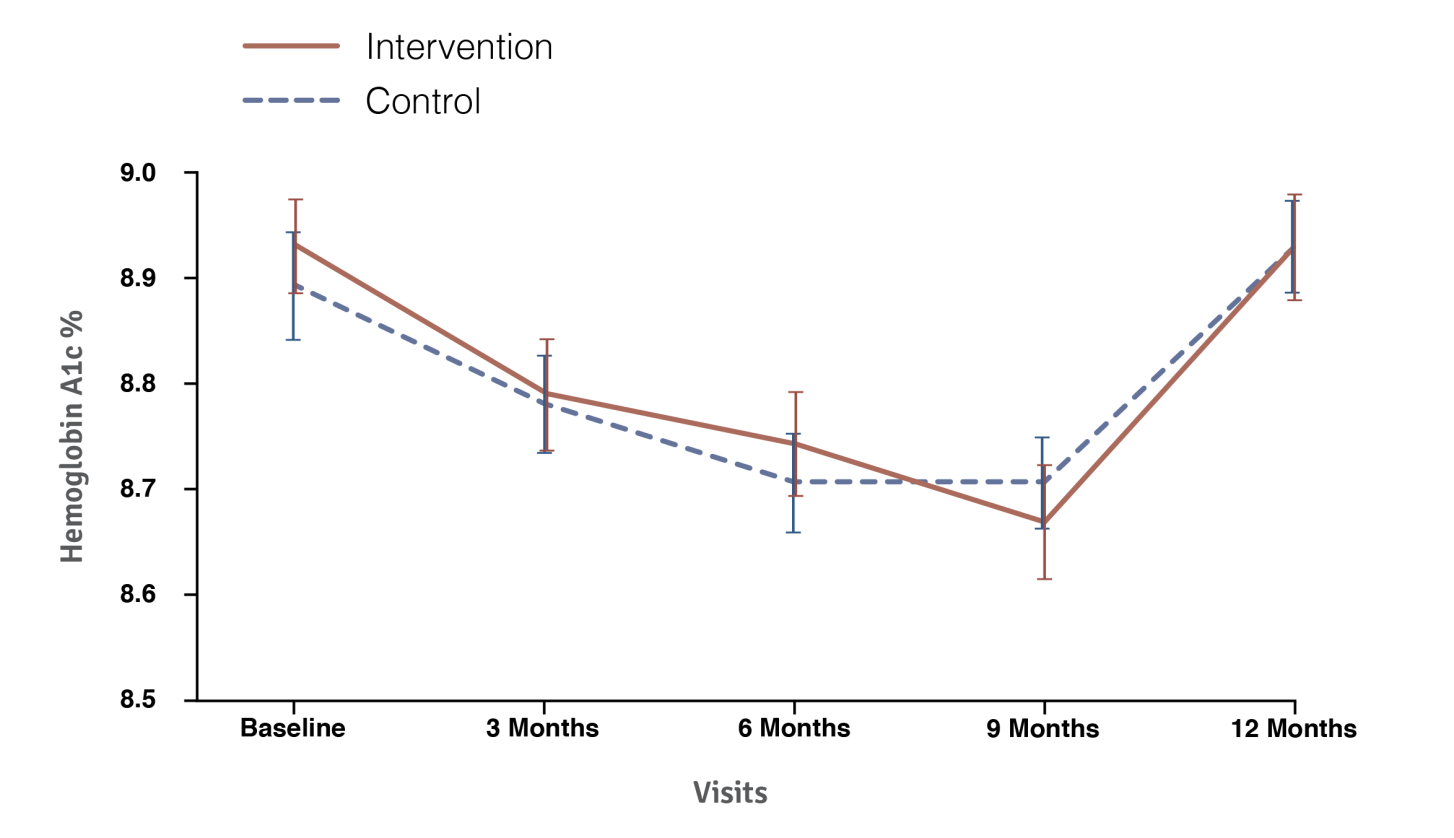
Mean hemoglobin A_1c_ values for the intervention and control groups from baseline to 12 months.

There were no significant differences in HbA_1c_ between the intervention and control groups over the duration of the 12-month trial (*P*=.99). Both groups demonstrated diminution in HbA_1c_ up to the 9-month time point, after which both experienced a subsequent increase to preintervention HbA_1c_ levels. This diminution speaks to study effects from the trial and demonstrates the importance of the control group. At trial conclusion, the intervention and control group displayed a mean HbA_1c_ of 8.96 (±1.3) and HbA_1c_ of 8.96 (±1.2), respectively (Figure 3).

Between group analyses also showed no significant improvements in any of the predefined secondary outcomes between the intervention and control groups (Table 3).

**Table 3:** Secondary outcome measures.

Outcome measures		Intervention		Control		*P* value (between-group)
		Baseline	12 months	Baseline	12 months	
Mild hypoglycemic events^a^, mean (SD)		10 (8.2)	11.52 (10.7)	8.49 (9.6)	7.54 (7.7)	.047
Severe hypoglycemic events^b^, mean (SD)		0.23 (0.6)	0.16 (0.4)	0.41 (1.3)	0.48 (1.2)	.13
Self-monitoring blood glucose^a^, mean (SD)		3.98 (1.6)	3.49 (1.8)	3.55 (1.6)	3.39 (1.5)	.42
Number of adjustments to regimen^b^, mean (SD)		1.85 (2.3)	1.77 (2.7)	2.08 (3.4)	1.10 (1.3)	.25
SCI score^c^, mean (SD)		35.73 (4.6)	35.42 (5.0)	36.07 (5.4)	35.57 (6.4)	.81
**DQOLY^d^subscale scores, mean (SD)**						
	Impact of Symptoms	3.58 (1.7)	3.33 (1.7)	3.55 (1.8)	3.16 (1.6)	.15
	Impact of Treatment	2.76 (2.3)	2.53 (2.1)	2.73 (2.0)	2.28 (2.2)	.51
	Impact on Activities	3.00 (2.2)	2.96 (3.0)	3.04 (2.8)	3.42 (3.0)	.72
	Parental Issues	5.13 (3.3)	5.20 (3.6)	5.12 (3.1)	4.67 (3.6)	.71
	Worries About Diabetes	6.83 (5.5)	6.84 (5.8)	6.51 (5.8)	4.81 (5.0)	.17
	Health Perception	2.00 (0.7)	1.96 (0.7)	1.90 (0.6)	2.10 (0.6)	.50
**DFRQ^e^overall and subscale scores, mean (SD)**						
	General Health Domain	12.76 (2.2)	13.70 (2.4)	12.53 (2.1)	13.31 (2.8)	.60
	Social Presentation Domain	8.62 (1.6)	8.86 (1.5)	8.81 (1.5)	9.08 (1.4)	.38
	Regimen Domain	13.90 (2.4)	14.60 (2.1)	13.61 (2.5)	14.40 (2.7)	.64
	Total DFRQ score	35.29 (4.9)	37.16 (4.3)	34.94 (4.6)	36.79 (5.7)	.78

^a^Average number over 50 days prior to study clinic appointment.

^b^Average number between study clinic appointments (typically 90 days).

^c^SCI: Self-Care Inventory, a 14-item questionnaire using 6-point scale (1 to 5, and “not applicable” option) to measure adherence to treatment recommendations. Overall score ranges from 10 to 50.

^d^DQOLY: Diabetes Quality of Life for Youth questionnaire, a 22-item questionnaire measuring quality of life, split across 6 subscales. Subscales use an inverted 5-point Likert scale (0 to 4), with the exception of the Health Perception subscale, which uses an inverted 4-point scale (1 to 4). Higher scores are associated with poorer quality of life; possible subscale scores range from 1 to 4 (Health Perception), 0 to 12 (Impact of Symptoms, Impact of Treatment, Parental Issues), 0 to 20 (Impact on Activities), and 0 to 28 (Worries About Diabetes).

^e^DFRQ: Diabetes Family Responsibility Questionnaire, a 17-item questionnaire measuring adolescent-guardian interaction around care, split across 3 subscales. All subscales use a 3-point scale (1 to 3). Higher scores are associated with increased adolescent involvement in care. Overall score ranges from 17 to 51; subscales range from 7 to 21 (General Health Domain), 4 to 12 (Social Presentation Domain), and 6 to 18 (Regimen Domain).

### Exploratory Analyses

**Figure 4:**
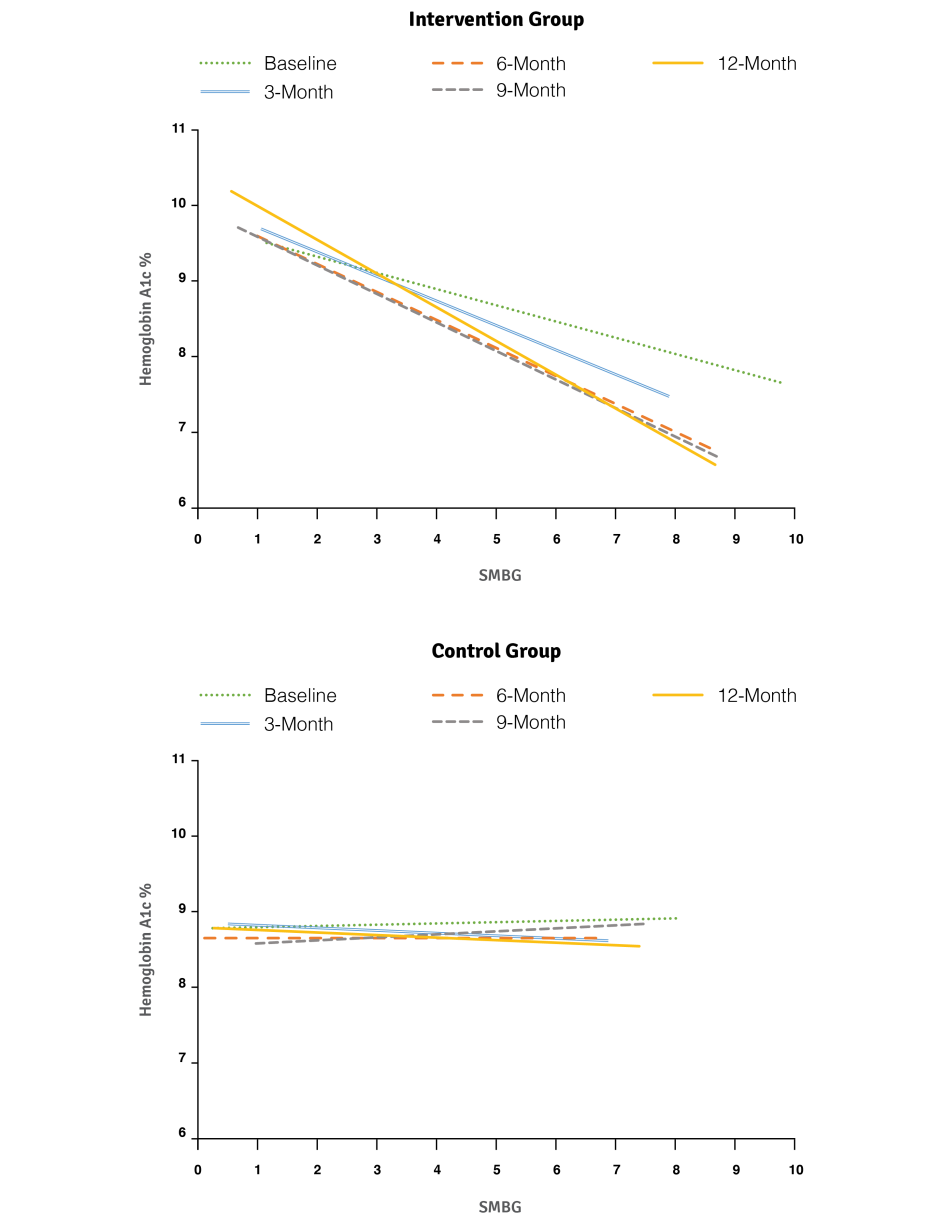
Regression analysis for self-monitoring of blood glucose (SMBG) and hemoglobin A_1c_.

Using all available data at each time point, we performed additional analyses to identify potential relationships between measured clinical outcomes, both within and between the intervention and control groups. Figure 4 shows a significant relationship between increased SMBG and improved HbA_1c_ in the intervention group at baseline, which strengthened over time, specifically when comparing 9-month (*P*=.002) and 12-month visits (*P=*.008) with baseline. This relationship was not observed in the control group at any time point (n between 32 and 46 for comparison).

In further exploratory analyses, we identified a subgroup of patients with a frequency of SMBG of 5 or more per day at 12 months within both the intervention (n=8) and control (n=5) groups. This threshold was chosen because it is a commonly recommended daily SMBG target in The Hospital for Sick Children diabetes clinic, and this group represented a population of users who were actively engaged with daily SMBG at the end of the trial. No significant difference in daily SMBG was noted between the control subgroup (mean 7.02, SE 0.57) and the intervention subgroup (mean 6.32, SE 0.45) at baseline *(P=*.34). Similarly, at 12 months, there was also no significant difference in SMBG frequency between participants in the control (mean 6.24, SE 0.57) and intervention (mean 6.33, SE 0.45) subgroups *(P=*.90).

**Figure 5:**
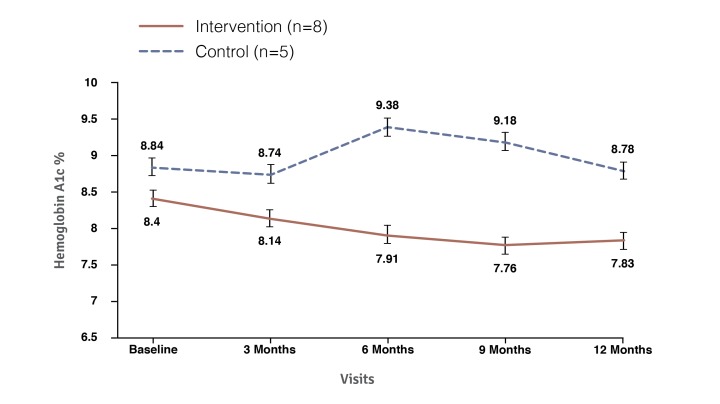
Longitudinal mean hemoglobin A_1c_ for intervention and control participants with 12-month self-monitoring of blood glucose of 5 or more per day.

HbA_1c_ did not significantly differ between the 2 subgroups at baseline (control mean 8.84%, SE 0.27% vs intervention mean 8.40%, SE 0.21%; *P=*.21). However, as shown in Figure 5, at the 6-month time point, users in the intervention subgroup demonstrated a significantly lower HbA_1c_ when compared with the controlled subgroup (*P*<.001), a difference that persisted for the remainder of the trial (9 months, *P*<.001; 12 months, *P*=.008). Furthermore, the *bant* subgroup demonstrated an overall improvement in HbA_1c_ of 0.58% (*P*=.02), while the parallel subgroup in the control arm experienced no significant change in HbA_1c_ (decrease of 0.06%, *P=*.84).

In addition to the subset with SMBG of 5 or more per day, we also conducted subgroup analyses for insulin regimen (insulin pump vs insulin injections), as well as baseline HbA_1c_ levels (participants with baseline HbA_1c_ ≥9.0% vs <9.0%); however, no statistically significant differences were noted.

### bant Usage Data

To assess use of *bant* over the course of the study, engagement levels were established. Given that the app was designed to facilitate daily SMBG and self-management activities, the engagement threshold levels were based on the total number of days that a user wirelessly uploaded blood glucose readings to *bant* over 12 months. As Table 4 shows, 4 levels of engagement (very low, low, moderate, and high) were used, where the highest engagement level was defined by a data upload frequency greater than 3 out of 7 days.

**Table 4:** Engagement thresholds, determined by the frequency of reading uploads, during the 12-month trial (n=46).

Engagement levels	Definitions	Injections (n)	Insulin pump (n)	Total (n)	% of all participants within each threshold
Very low	Less than 1 of 14 days	9	8	17	37
Low	Less than 1 of 7 days	6	7	13	28
Moderate	Less than 3 of 7 days	5	7	12	26
High	3 of 7 days or more	3	1	4	9
Total		23	23	46	100

Overall, usage of *bant* showed a significant interaction with SMBG *(P=*.03), with users in the high-engagement group having a significantly higher frequency of SMBG throughout the trial than users with either low *(P=*.004) or very low engagement *(P=*.02). Further analyses demonstrated no significant association between *bant* usage and any other clinical outcomes.

### Satisfaction

**Figure 6:**
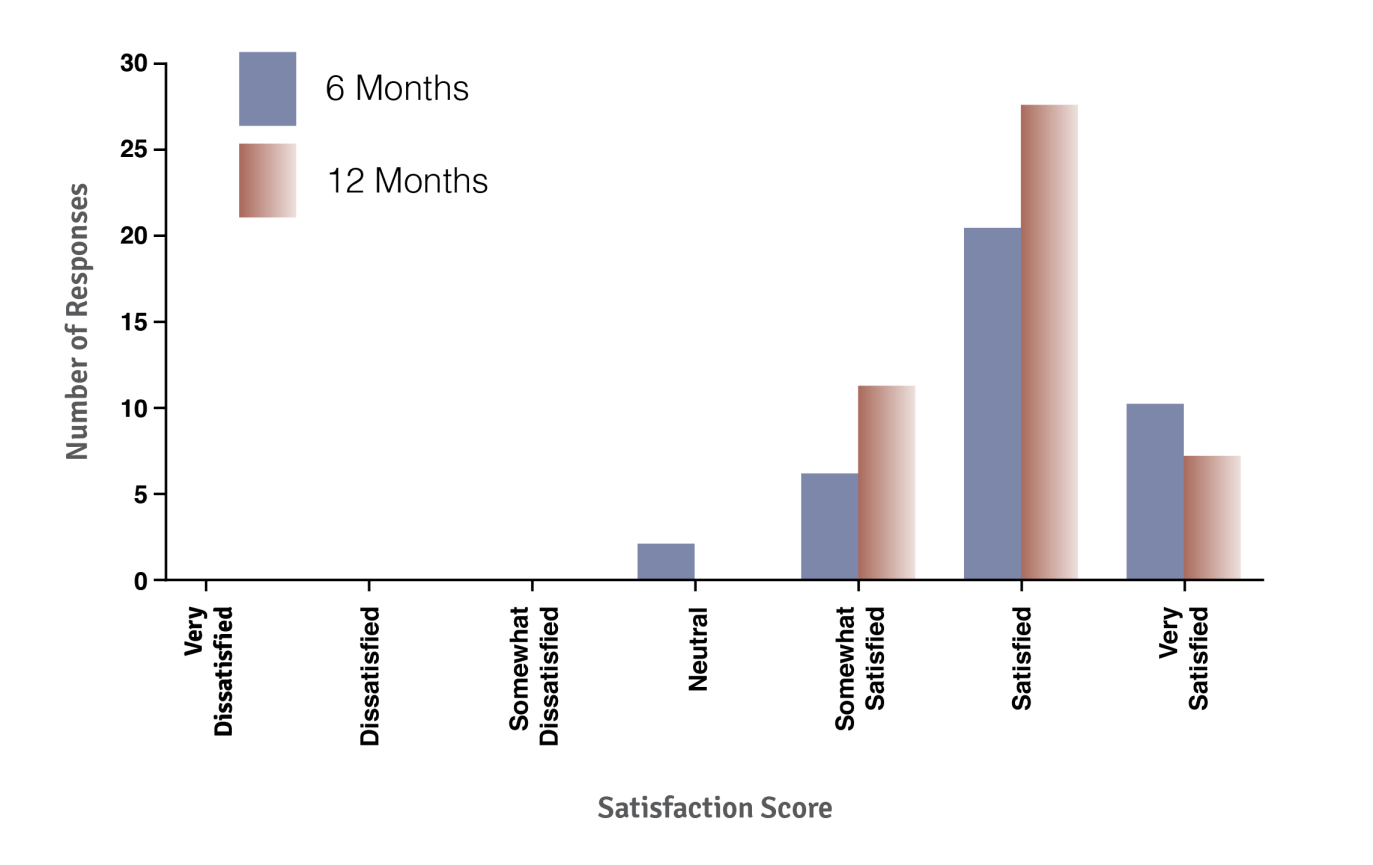
Overall satisfaction with *bant* at the 6- and 12-month time points.

Participants reported high levels of satisfaction with *bant* throughout the trial (Figure 6). At 6 and 12 months, 79% (30/38) and 76% (34/45) of participants reported being “satisfied” or “very satisfied” with *bant*, respectively. In addition, 96% (43/45) of respondents reported that they would continue to use *bant* if it were available to them outside of the trial.

We also asked users to rank the features of *bant* according to their perceived usefulness in assisting with daily self-management of type 1 diabetes. Overall, the trending feature was ranked as the most useful component of *bant* by 45% (20/44) of respondents. This was followed by the logbook, which was ranked most useful by 14% (6/44), and the app home page (which displays current readings with respect to target range), which was ranked most useful by 11% (5/44).

## Discussion

### Principal Findings

The aim of this 12-month RCT was to evaluate the effectiveness of *bant*, an mHealth app for the self-management of type 1 diabetes among adolescents. Although satisfaction was high across the duration of the trial, with a defined subset of users regularly accessing and using *bant*, overall we noted no significant improvements in primary or secondary outcomes.

While primary clinical outcomes remained unchanged, a post hoc exploratory analysis provided additional insights. A significant and strengthening relationship between increased SMBG and improved HbA_1c_ was observed exclusively in the intervention group (Figure 4), suggesting that *bant* users may have better used their SMBG data for the self-management of type 1 diabetes. This finding was reinforced by a subgroup analysis conducted on participants who were taking 5 or more SMBG a day at their 12-month visit. Users in this *bant* subgroup (n=8) demonstrated significant improvements in HbA_1c_ when compared with the parallel control subgroup (n=5), with a statistically and clinically significant decrease in HbA_1c_ of 0.58% over the trial duration. Thus, it is possible that, for those users who were testing frequently, *bant* enabled better self-management of diabetes, resulting in an improved HbA_1c_, when compared with usual care.

To identify any factors that may have influenced the overall trial results, we conducted several secondary analyses, including the characteristics of the study population and potential trial design artifacts. This RCT purposefully targeted adolescents who were experiencing difficulty in managing their diabetes, as defined by sustained HbA_1c_ values between 8.0% and 10.5%, who might benefit greatly from enhanced self-management skills and motivation. However, it is possible that, by extending the HbA_1c_ inclusion range to 10.5%, patients whose poor glycemic control was caused by multiple complex factors, requiring support beyond the scope of the *bant* features, were detrimentally included in the study. While the study was not powered to look at subgroups, we conducted secondary analysis on users with a baseline HbA_1c_ of 9.0% or more and HbA_1c_ below 9.0%. The results showed no significant changes in glycemic control over the trial duration within either subgroup, suggesting that baseline HbA_1c_ was not predictive of *bant* ’s effectiveness.

In addition, with equal numbers of participants on an insulin pump versus insulin injections, it was also possible that the insulin regimen may have affected clinical outcomes. However, secondary subgroup analysis was conducted, which showed no significant impact of *bant* on glycemic control, or any other clinical outcomes, in either the pump or the injector group.

We also hypothesized that a poorly motivated participant population could have resulted in the lack of improvement in clinical outcomes. However, the Readiness to Change Survey data showed that, on average, the intervention and control groups were classified in similar stages of change at baseline—including the “preparation” stage of change (for increased SMBG), associated with individuals who are ready to implement a plan of action to improve their health outcomes [45]. This observation, paired with the previously discussed subgroup results, suggests that the lack of significance found during primary analysis was likely not due to the demographics of our study population.

**Figure 7:**
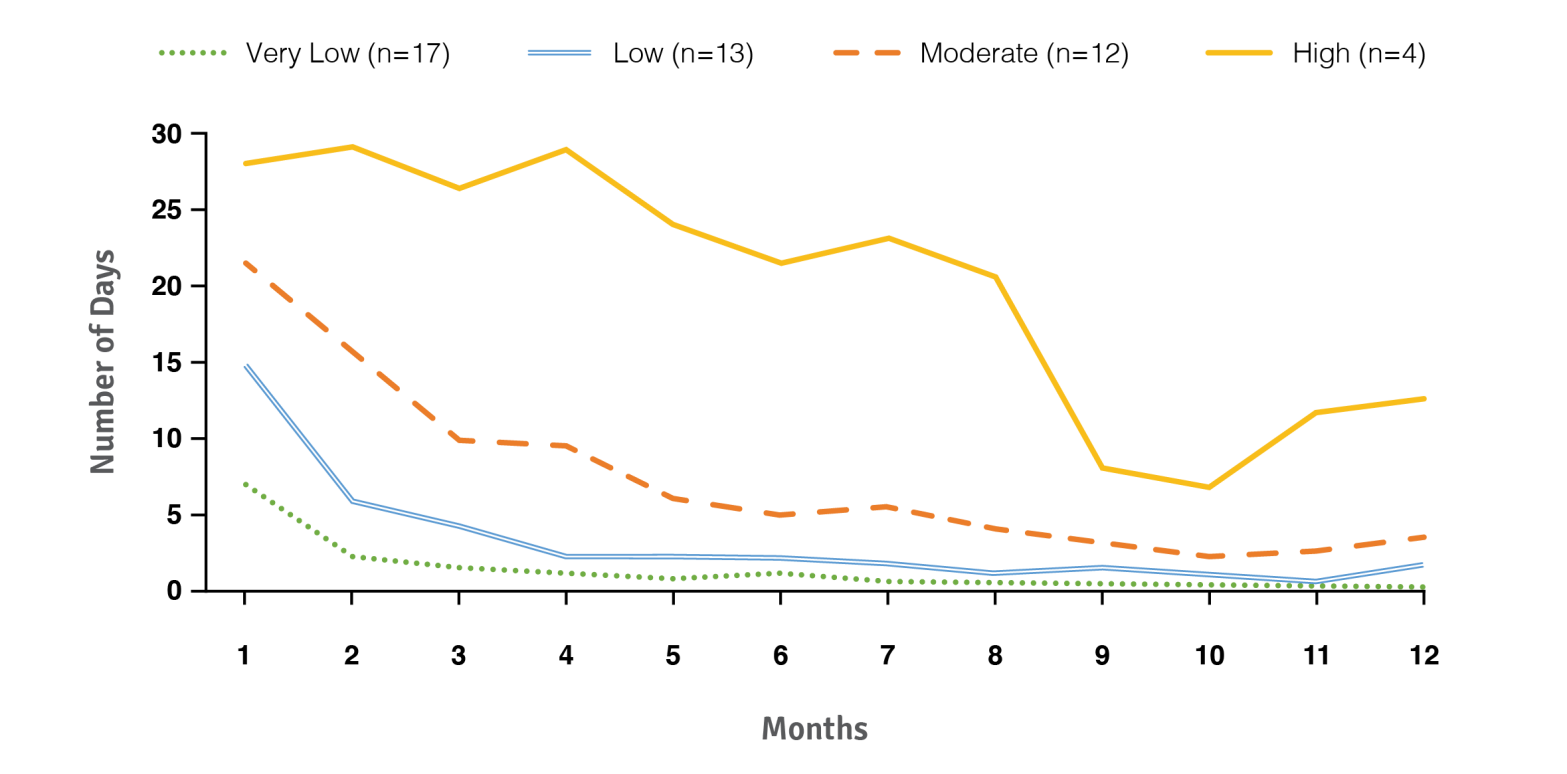
Number of times (measured as days per month) users uploaded blood glucose data to *bant* across the study duration.

The *bant* usage data (Figure 7) indicated that, for many of the participants, the regular use of the app extended beyond the average 3- to 5-week engagement period reported by other mobile app industries [48,49]. This finding is in accord with the satisfaction data (Figure 6), and implies that future versions of *bant* may also be well used. However, over the 12-month trial duration, only 35% of users (n=16) wirelessly uploaded blood glucose data to *bant*, on average, once or more per week (Table 4). Given that the key self-management features of *bant* require blood glucose data, it can be inferred that usage of the app is dependent on users uploading data in the first place. There are 2 key factors that may have resulted in the low frequency of data uploads and are recognized limitations of the currently assessed system: (1) providing patients with a secondary mobile phone, and (2) the functionality of BluGlu.

First, participants in the intervention arm were given *bant* on a study-provided mobile phone, rather than installing the app directly on their own personal devices. While this was intentional, ensuring that all participants had equitable access to the iOS app, recent data indicate that many of these adolescents likely already owned a mobile phone, and therefore the addition of the study phone may have placed an unanticipated burden on the participant [16]. A key strength of mHealth is the ability to capture data and provide feedback for users via their personal devices, which are embedded into their daily routines. Providing the intervention on an additional secondary phone may have defeated the concept of embedded health interventions, as it is likely that many participants may not have wanted, or be able, to carry 2 mobile phones for 12 months.

Interestingly, in the 2011 study (n=20), *bant* elicited a significant increase in SMBG [32]. It can be hypothesized that at this time there were lower levels of mobile device penetration among adolescents, and the novelty of having an iPhone would likely compel participants to use the device as a primary phone. Future studies should deploy mHealth apps directly onto personal mobile phones in order to improve usage and facilitate seamless integration into daily life.

Second, we developed the RCT version of *bant* before the emergence of Bluetooth-enabled blood glucose meters. As such, we developed our own adapter, BluGlu, to facilitate the wireless upload of data from blood glucose meters to *bant*. However, this adapter was only compatible with the OneTouch UltraMini blood glucose meter. Throughout the study, a subset of participants continued to use additional blood glucose meters, often of a different brand. Therefore, it is possible that asking participants to use an external adapter, which only worked with one particular blood glucose meter, hindered the full integration of *bant* into their existing diabetes management routines. Over the duration of the RCT, several Bluetooth-enabled meters came onto the market, enabling a “plug and play” environment. A future consideration is to enable an open ecosystem so that users can have the option of using whichever wireless blood glucose meter suits their specific needs; this flexibility, along with no longer needing an external adapter, may improve use of mobile self-management platforms.

Another aspect that should be considered is the role of caregivers in the self-management activities adolescents perform using mobile tools. One of the key themes that emerged during the initial user-centered design of *bant* was the desire for adolescents to share their diabetes-related information with parents, peers, and clinic staff [32]. A recent literature review by Deacon et al suggested that mobile interventions that encourage data collection as well as clinician feedback may be more successful at decreasing HbA_1c_ [50]. *bant* included a feature that allowed users to store their data in TELUS health space, a secure personal health record that allowed them, if desired, to share their data with those within their circle of care. It was not possible to gather data around the use of this feature; however, based on interactions with participants, it is likely that *bant* was used as a stand-alone self-management tool. The next iteration of *bant* should explore adding features that easily enable adolescents to receive feedback from caregivers and approaches that integrate the app into routine clinical care.

The study results illustrate the importance of rigorously evaluating mHealth apps, not only for understanding the impact on clinical outcomes and user engagement, but also for assessing the methods used to evaluate these tools. While traditional RCTs have been considered as the “gold standard” for evaluation of interventions, a recent review by Pham et al emphasized that RCTs may not be best suited for the evaluation of rapidly evolving software interventions [23]. Traditional RCTs are lengthy (average 5.5 years from enrollment to publication), expensive, and follow a rigid protocol that might not consider the sociotechnical, personal, and social components of mHealth implementation [23]. Perhaps more important, in the context of apps, they restrict the intervention to a static design and limit the ability to dynamically tailor the intervention based on unique needs of individuals. Future evaluations of *bant* and other mHealth apps should consider use of alternative research methodologies or adaptive RCT study designs [23]. For example, mPower, one of the first ResearchKit (Apple Inc) -enabled observational mHealth iOS app trials, demonstrated a completely electronic and in-app consent, enrollment, and study intervention, and 48,104 participants downloaded the app within the first 6 months of the public launch. Participants completed questionnaires at predetermined time intervals and used the native functionality of the mobile phone and its sensors to quantify symptoms of Parkinson disease (eg, tapping the screen to evaluate dexterity) [49]. Additionally, the Sequential Multiple Assignment Randomized Trial (SMART) adaptive study design enables the identification of the most effective intervention component sequencing strategy, by evaluating outcomes at predetermined time intervals. In this case, we could allocate groups to a specific combination of *bant* features and, based on the outcomes at a predetermined time point, alter the intervention according to a feature sequencing protocol, allowing us to rapidly converge on optimal intervention designs based on unique patient trajectories [51]. The Multiphase Optimization Strategy adaptive study design ensures the effectiveness of an intervention’s individual components and allows for incremental optimization of an intervention, prior to a full-scale RCT [51].

### Conclusions

Robust and scalable research methods, coupled with adaptive RCT study designs, have the potential to reshape mHealth research. These approaches can enable the rigorous evaluation of apps in a more timely manner, while facilitating the rapid and iterative development of an intervention, keeping pace with the rapidly and continuously evolving mHealth landscape.

While adolescents are increasingly accessing technologies to support the self-management of type 1 diabetes, the impact of these tools on clinical outcomes remains unclear. Although this RCT found no changes in primary and secondary outcomes, exploratory analysis demonstrated improved HbA_1c_ among *bant* users who tested blood glucose more frequently. This suggests that these users gained insights around their SMBG data, which may have led to positive changes in their self-management behavior. Overall satisfaction levels were high, suggesting that app users found utility in *bant*, specifically in features related to management of out-of-range blood glucose trends. The next iteration of *bant* will explore features that diminish barriers to use, enable deployment directly to personal mobile phones, are integrated into the daily clinical routine, and enable more frequent feedback from caregivers. Future evaluations of apps for diabetes self-management may also benefit from exploring methodologies that allow for more practical, scalable, and robust evaluation, given the challenges associated with rapidly evolving technology and consumer expectations.

## Appendix

Multimedia Appendix 1 CONSORT-EHEALTH checklist V1.6.1.

Multimedia Appendix 2 Overview of the main pages of bant.

Multimedia Appendix 3 Participant Management Questionnaire.

## Abbreviations

ANOVA: analysis of variance
DFRQ: Diabetes Family Responsibility Questionnaire
DQOLY: Diabetes Quality of Life for Youth
HbA1c: hemoglobin A1c
RCT: randomized controlled trial
SMART: Sequential Multiple Assignment Randomized Trial
SMBG: self-monitoring of blood glucose
