# Supplementary material for: A Mobile App for the Self-Management of Type 1 Diabetes Among Adolescents: A Randomized Controlled Trial
Source: JMIR Mhealth Uhealth. 2017 Jun 19;5(6):e82. doi: 10.2196/mhealth.7336 (PMC5495971; doi:10.2196/mhealth.7336)

The screen shots below provide an overview of the main pages *bant* as well as the trending features: a) home page, b) trends, c) *bant* book, d) rewards, e) *banter*, and f) the trending wizard.

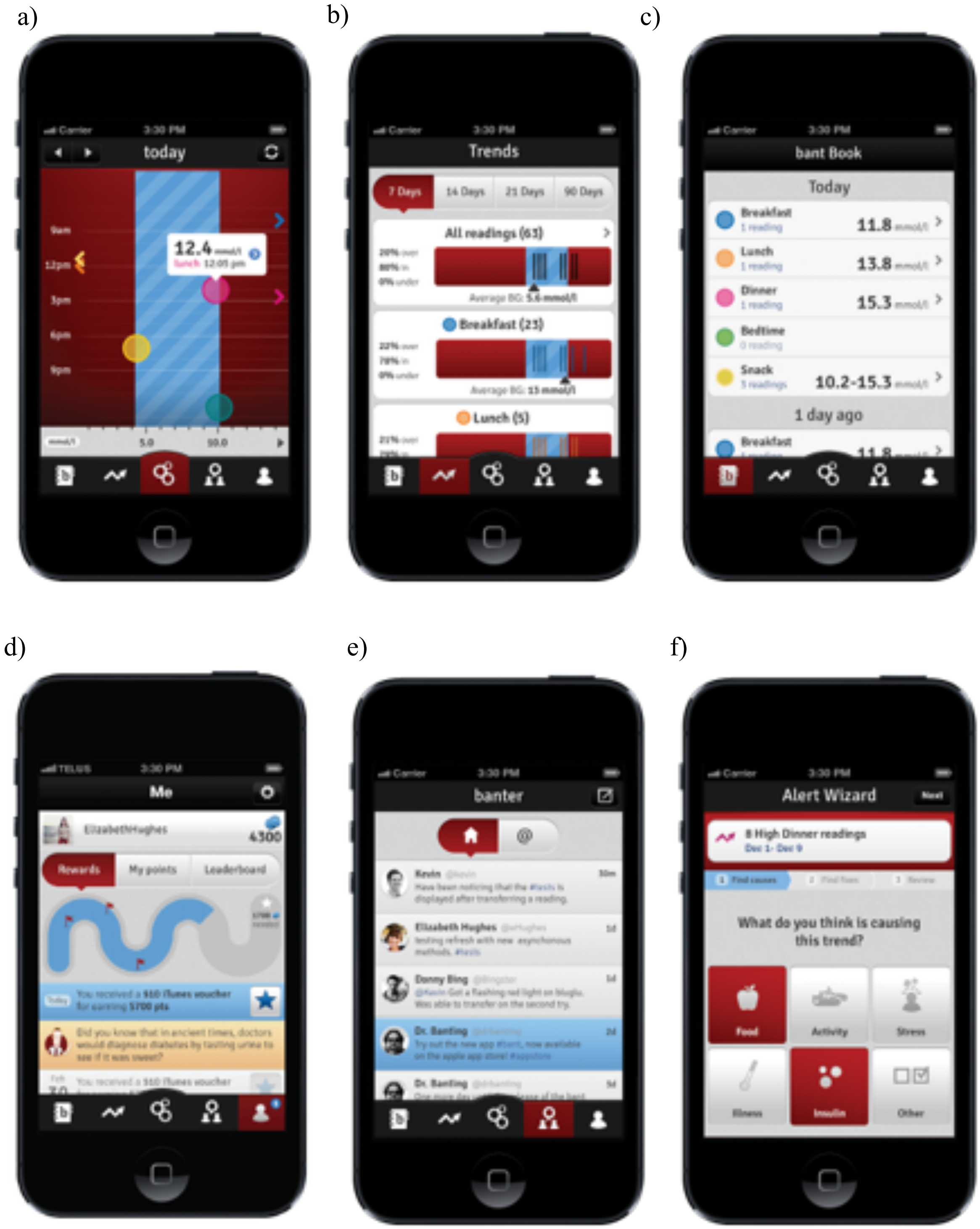

Supplement: Multimedia Appendix 2 [file mhealth_v5i6e82_app2.pdf]
