# Supplementary material for: A Mobile App for the Self-Management of Type 1 Diabetes Among Adolescents: A Randomized Controlled Trial
Source: JMIR Mhealth Uhealth. 2017 Jun 19;5(6):e82. doi: 10.2196/mhealth.7336 (PMC5495971; doi:10.2196/mhealth.7336)
Supplement: Multimedia Appendix 3 [file mhealth_v5i6e82_app3.pdf]

Appendix B – Participant Management Questionnaire

The Transtheoretical Model (TTM) of behaviour change is an integrative model which uses defined stages (pre-contemplation, contemplation, preparation, action and maintenance) to explain how individuals modify their health behaviours [44, 45]. Using these TTM stages of change, we measured subject's readiness to change their: 1) frequency of blood glucose checks, and 2) frequency of self-initiated regimen adjustments.

All subjects were given the Participant Management Questionnaire (below) and asked to select 1 out of 6 statements that best described their thoughts around changing a health behaviour. Statement 1 and 2 both corresponded to the pre-contemplation stage, with all other statements corresponding to the remaining 4 stages. In addition, using a 10-point Likert scale (with 10 being the highest score), subjects were also asked to rate how confident and motivated they were to make a change to each health behaviour. The Participant Readiness Survey was distributed to all subjects at baseline only.

Participant Management Questionnaire

Study Title: Assessment of an Electronic Self-Management Tool on Glycemic Control in Teens with Type 1 Diabetes

Unique Study ID –

Version Date: 11/06/2013

Question #1
Please read each of the following 5 statements about yourself. Check the box regarding the **one** statement that best describes how you think about checking blood sugars:

- ☐ 1) I have not even thought of checking my blood sugars more often.
- ☐ 2) I don't believe that I need to check my blood sugars more often.
- ☐ 3) I might need to check my blood sugars more often, and I have been thinking about what to do about it.
- ☐ 4) I am ready to do something about checking my blood sugars more often, but haven't yet.
- ☐ 5) I have actively been trying to check my blood sugars more often.
- ☐ 6) I have successfully made changes and now check my blood sugars more often.

For Question 2 and 3 circle **one** number. Please use the scale of 1-10, with 10 being the highest.

Question #2
How motivated are you to check your blood sugars more frequently?

1 2 3 4 5 6 7 8 9 10

Question #3
How confident are you that you can check your blood sugars more frequently?

1 2 3 4 5 6 7 8 9 10

Question #4
Please read each of the following 5 statements about yourself. Check the box regarding the **one** statement that best describes how you think about making changes to your diabetes regimen (insulin doses and/or diet):

- ☐ 1) I have not even thought of making more changes of my own to my diabetes regimen.
- ☐ 2) I don't believe that I need to make more changes of my own to my diabetes regimen.
- ☐ 3) I might need to make more changes of my own to my diabetes regimen, and I have been thinking about how to do this.
- ☐ 4) I am ready to make more changes of my own to my diabetes regimen, but haven't yet.
- ☐ 5) I have actively been trying to make more changes of my own to my diabetes regimen.
- ☐ 6) I have now made more changes of my own to my diabetes regimen.

For Question 5 and 6 circle **one** number. Please use the scale of 1-10, with 10 being the highest.

Question #5
How motivated are you to make more frequent changes of your own to your diabetes regimen (insulin doses and/or diet)?

1 2 3 4 5 6 7 8 9 10

Question #6
How confident are you that you can make more frequent changes of your own to your diabetes regimen (insulin doses and/or diet)?

1 2 3 4 5 6 7 8 9 10
